# An Investigation of the Acute Effects of Oligofructose-Enriched Inulin on Subjective Wellbeing, Mood and Cognitive Performance

**DOI:** 10.3390/nu7115441

**Published:** 2015-10-28

**Authors:** Andrew P. Smith, David Sutherland, Paul Hewlett

**Affiliations:** Centre for Occupational and Health Psychology, School of Psychology, Cardiff University, 63 Park Place, Cardiff CF10 3AS, UK; d.sutherland@abdn.ac.uk (D.S.); phewlett@cardiffmet.ac.uk (P.H.)

**Keywords:** Oligofructose-enriched inulin, cognition, memory, mood

## Abstract

Inulin is a natural food component found in many plants that are part of the human diet (e.g., leeks, onions, wheat, garlic, chicory and artichokes). It is added to many foods and is used to increase dietary fibre, replace fats or carbohydrates, and as a prebiotic (a stimulant of beneficial bacteria in the colon). Oligofructose, which is also present in these foods, produces similar effects and most research has used a combination of these products. A previous study (Smith, 2005) investigated the effects of regular consumption of oligofructose-enriched inulin on wellbeing, mood, and cognitive performance in humans. The results showed that oligofructose-enriched inulin had no negative effects but that it did not improve wellbeing, mood, or performance. The aim of the present study was to examine the acute effects of oligofructose-enriched inulin (5 g) over a 4 h period during which the participants remained in the laboratory. A double blind placebo (maltodextrin) controlled study (*N* = 47) was carried out with the order of conditions being counterbalanced and the two sessions a week apart. On each test day mood and cognitive performance were assessed at baseline (at 8:00) and then following inulin or placebo (at 11:00). Prior to the second test session (at 10:30) participants completed a questionnaire assessing their physical symptoms and mental health during the test morning. The inulin and placebo were provided in powder form in 5 g sachets. Volunteers consumed one sachet in decaffeinated tea or decaffeinated coffee with breakfast (9:00). Questionnaire results showed that on the day that the inulin was consumed, participants felt happier, had less indigestion and were less hungry than when they consumed the placebo. As for performance and mood tasks, the most consistent effects were on the episodic memory tasks where consumption of inulin was associated with greater accuracy on a recognition memory task, and improved recall performance (immediate and delayed). Further research is required to identify the mechanisms that underlie this effect with glucose metabolism being one candidate.

## 1. Introduction

There has been considerable recent interest in the brain-gut axis which reflects the bi-directional signaling between the gastrointestinal tract and the brain [1]. The brain-gut axis is regulated at the neural, hormonal and immunological level [2]. Research has investigated how bacterial colonization influences behaviour [3,4,5] and these effects have been shown to involve the vagal pathways [6] and neurotransmitter systems [7]. One method of changing gut flora is to use prebiotics to stimulate beneficial bacteria in the colon. Inulin has been shown to be a prebiotic [8,9] but also has other properties that may lead to changes in behaviour. Inulin is a natural food component found in many plants that are part of the human diet (e.g., leeks, onions, wheat, garlic, chicory and artichokes). For commercial purposes it is extracted from the chicory root by diffusion in hot water. It is added to many foods and is used to increase dietary fibre, replace fats or carbohydrates. Research has shown that inulin produces its most beneficial effects when combined with a similar compound, oligofructose, and most of the published studies have used this combination.

Inulin has been widely studied (a pubmed search reveals over 10,000 articles) but there has been little research on its behavioural effects. Animal research [10] suggests that ingestion of inulin may lead to improved cognitive performance. This study examined the behavioural and cognitive effects of oligofructose-enriched inulin at doses of 5% and 10% in the diet, orally ingested daily during two weeks, using a functional observational battery (FOB) and the light extinction test in male Wistar rats. Control rats received a standard diet and were tested in the same test situations. The behavioural effects were assessed two days before and 14 days after the beginning of the treatment period and the cognitive effects were investigated after the administration period by lever-pressing activity and learning discrimination using the light extinction test paradigm. In general, the study demonstrated that oligofructose-enriched inulin at 5% in the diet, and particularly at 10% in the diet, increased relaxation but also stimulated and increased the general activity and interest of the rats to the test environment. In addition, both doses of oligofructose-enriched inulin showed significant effects on learning discrimination in male rats, in comparison with the control diet. These results suggest that oligofructose-enriched inulin, particularly at the dose of 10%, improves cognitive performance and the well-being of male rats. The high doses used in this study would not be ingested by humans and most research has used doses of less than 15 g due to the increase in digestive symptoms with high doses.

A previous study [11] tested the hypothesis that oligofructose-enriched inulin regulates the digestive system so as to produce a state of improved psychological well-being (improved energy, mood, and cognitive function). That study investigated the effects of regular consumption of 10 g inulin over a 14 day period. This time period was used because it was hypothesised that inulin would lead to prebiotic changes in gut flora which would lead to reduced fatigue due to the production of short chain fatty acids and the elimination of toxins. The results showed that inulin had no negative effects but that it did not improve well-being, mood, or performance.

One of the major problems with studies of free living individuals is the lack of control over what the volunteers consume. In addition it is impossible to separate acute effects from those due to possible longer term changes in the gut flora. The aim of the present study was, therefore, to determine the acute effects of inulin added to a breakfast of cereal and toast, with tea or coffee on cognitive performance and mood over a 4 h period in the laboratory. Such a study eliminates prebiotic effects because they take a much longer time period to develop. However, inulin has been shown to produce rapid changes in physiology that could change mood and cognition through other mechanisms [12].

## 2. Methods

The study was carried out with the approval of the ethics committee, School of Psychology, Cardiff University, and with the informed consent of the volunteers.

### 2.1. Design

A cross-over design was used with half the participants having the inulin on the first visit and the placebo on the second (with one week between visits), and the other half having the placebo and inulin conditions in reverse order.

### 2.2. Participants

Volunteers for the study were screened prior to inclusion using the same criteria as a previous study of inulin and cognition [11]. They were excluded if self-reports showed that: (1) there was existing disease or participants were receiving medication; (2) if they were heavy smokers (>10 cigarettes a day); (3) if alcohol consumption was high (females >20 units a week; males > 30 units a week); and (4) if they were high fibre consumers (above 3rd quartile, as measured by a fibre questionnaire).

A sample size calculation showed that a medium effect size required a sample of 33 (G*Power [12]). Fifty participants were recruited; however three of these withdrew from the study. All three completed their first test session but then withdrew due to other commitments. The remaining sample size was sufficient to detect medium sized effects.

The sample consisted of 28 females and 19 males (mean age 23.0 years, range 19–30 years).

### 2.3. Procedure

After recruitment volunteers were given a familiarization session with the mood and performance tasks. On test days, participants fasted, consumed no caffeinated beverages and did not smoke prior to arriving at the laboratory. They reported to the laboratory at 8:00 and completed a baseline battery of computerised mood and performance tests lasting about 45 min. They were given a breakfast similar to their habitual breakfast at 9:00, and this consisted of breakfast cereal (cornflakes or rice crispies) and toast (with jam or marmalade). Participants were given de-caffeinated tea or de-caffeinated coffee to which the inulin or placebo were added. Before the second test session participants completed questionnaires relating to how they had recently been feeling over the past seven days and over the morning that day (10:30). The next test session was at 11:00. Only one test session was carried out as a longer period in the laboratory was not possible for some participants and would also have required additional feeding which may alter any effects of inulin.

### 2.4. Inulin/Placebo

The inulin and placebo were provided in powder form by ORAFTI (Tienen, Belgium) and volunteers consumed the contents of one sachet (5 mg) at breakfast (9:00) in a drink (decaffeinated tea or decaffeinated coffee). This dose was used because there is some evidence of digestive problems with larger doses.

### 2.5. Measures

Volunteers completed a battery of questionnaires [11] assessing their fatigue/energy, subjective mood, physical and mental health and fibre intake over the week prior to the test day. These were recorded to check that volunteers did not differ prior to placebo and inulin days.

At the end of the test session, volunteers completed a diary describing how they felt that morning. The main focus was on mood (feeling energetic, fatigued, stressed, anxious, depressed, happy), appetite and digestive problems. Responses were made on a 5-point scale from “Never” to “Very Often” or, in the case of digestive symptoms, were Yes/No answers.

At each test session (8:00, 11:00), volunteers completed the performance tests and mood ratings described below. This battery of tasks have been used since 1992 [13] and was based on established tests which had been used in other forms (e.g., paper and pencil). Key features of the presentation of the current tasks has been the use of an external response box and millisecond accuracy measurement of response times. The version used here had an external response box connected to the serial port of the computer. Presentation of stimuli and recording of response used a micro-chip mounted in the response box, the package was written in visual basic and produced by Cologic Ltd. (Chichester, UK). Tests from the battery have been used in over 100 published studies [14].

### 2.6. Mood

This was measured before and after the cognitive tests using 18 bi-polar visual analogue scales (e.g., Drowsy-Alert, Tense-Calm [15]) presented on the screen of an IBM compatible computer (Compaq, Harris County, TX, USA). An adjective was at each end of the screen and the participant moved a cursor along a line to indicate their current mood. Scores were from 1 (very left of the line) to 50 (very right of the line). Factor analysis has shown that the scales load on three dimensions: alertness, hedonic tone and anxiety.

### 2.7. Performance Tasks

A battery of tests were used that measure a range of functions. All of these tests were presented on an IBM compatible PC. These tests are known to be sensitive to changes in state [16,17,18,19,20].

### 2.8. Memory Tasks

#### 2.8.1. Free Recall Task

In this task [15] a list of 20 words were presented on the PC screen at a rate of one every 2 s. At the end of the list, the volunteer had 2 min to write down (in any order) as many of the words as possible (“immediate recall”). Towards the end of the test battery (approximately 40 min after the words were presented) the volunteers were again asked to write down as many as they could remember (“delayed recall”).

#### 2.8.2. Delayed Recognition Memory Task

This task [15] was at the end of the test session, and volunteers were shown a list of 40 words, which consisted of the 20 words shown at the start of the session plus 20 distracters. They had to decide as quickly as possible whether each word was shown in the original list or not and press the appropriate response key.

#### 2.8.3. Logical Reasoning Task

In this task [21] the participants were shown statements about the order of the letters A and B followed by the letters AB or BA (e.g., “A follows B: BA”). They had to read the statement and decide whether the sentence was a true description of the order of the letters. If it was, they pressed the “True” key on the response box; if it wasn't, they pressed the “False” key. The sentences ranged in syntactic complexity from simple active to passive negative (e.g., “A is not followed by B”). Volunteers carried out the task for 3 min.

#### 2.8.4. Semantic Processing Task

This test [22] measures speed of retrieval of information from general knowledge. Volunteers were shown a sentence and had to decide whether it was true (e.g., “canaries have wings”) or false (e.g., “dogs have wings”) and then press the appropriate response key The number completed in 3 min was recorded, as was accuracy.

#### 2.8.5. Spatial Memory Task

This test [15] measures ability to remember a sequence of lights. Volunteers were shown a sequence of lights with each light appearing in a different location (a point of a pentagon) and had to remember and repeat (by pressing buttons representing the locations) the order in which the lights were presented. The number of trials completely correct and the % correct key presses were recorded.

### 2.9. Psychomotor Tasks: Simple Reaction Time Tasks

Two simple reaction time tasks [15] were performed. One of them had a variable fore-period (1–8 s), whereas in the other the time between the warning signal and presentation of the target was fixed (2 s). In both tasks a box was displayed on the screen and this was followed by a square (the target) being presented in the middle of the box. The participant had to press a key on the response box as soon as the square was detected, and following this another box was presented. These tasks lasted for 3 min each.

### 2.10. Selective Attention Tasks

#### 2.10.1. Focused Attention Task

This choice reaction time task [23] measured various aspects of performance. In this task target letters appeared as upper case A’s and B’s in the centre of the screen. Participants were required to respond as quickly and as accurately as possible to the target letter presented in the centre of the screen, ignoring any distracters presented in the periphery. The correct response to A was to press a key with the forefinger of the left hand while the correct response to B was to press a different key with the forefinger of the right hand. Prior to each target presentation three warning crosses were presented on the screen, the outside crosses were separated from the middle one by either 1.02 or 2.60 degrees. The crosses were on the screen for 500 ms and were then replaced by the target letter. The central letter was either accompanied by: (1) nothing; (2) asterisks; (3) letters which were the same as the target; or (4) letters which differed from the target. The two distracters presented were always identical and the targets and accompanying letters were always A or B. Participants were given ten practice trials followed by three blocks of 64 trials. In each block there were equal numbers of near/far conditions, A or B responses and equal numbers of the four distracter conditions. The nature of the previous trial was controlled. This test lasted approximately 6 min.

In this task several aspects of choice responses to a target were measured. The global measures of choice reaction time were mean reaction time and accuracy of response (percent correct) when the target was presented alone or when distracters were present. Long response times (>800 ms) were also recorded.

#### 2.10.2. Categoric Search Task

This task [23] was similar to the focused attention task previously outlined. Each trial started with the appearance of two crosses either in the central positions occupied by the non-targets in the focused attention task, *i.e.*, 2.04 or 5.20 degrees apart or further apart, located towards either left and right extremes of the screen. The target letter then appeared in place of one of these crosses. However, in this task participants did not know where the target would appear. On half the trials the target letter A or B was presented alone and on the other half it was accompanied by a distracter, in this task a digit (1–7). Again the number of near/far stimuli, A *versus* B responses and digit/blank conditions were controlled. Half of the trials led to compatible responses (*i.e.*, the letter A on the left side of the screen, or letter B on the right) whereas the others were incompatible. The nature of the preceding trial was also controlled. In other respects (practice, number of trials, *etc.*) the task was identical to the focused attention task. This task also lasted approximately 6 min. As in the focused attention task several aspects of choice responses to a target are measured. The global measures recorded were choice reaction time and accuracy of response when the target was presented alone in either near/far locations. Long response times (>1000 ms) were also recorded.

### 2.11. Sustained Attention Task: Repeated-Digits Vigilance Task

In this task [15] three-digit numbers were shown on the screen at the rate of 100 per minute. Each number was normally different from the preceding one by one digit but occasionally (8 times a minute) the same number was presented on successive trials. Participants had to detect these repetitions and respond as quickly as possible. The number of hits, reaction times for hits, and false alarms were recorded. The task lasted for 3 min.

### 2.12. Statistical Analysis

Analyses of the mood and performance data involved analyses of co-variance using the BMDP P2V programme [24]. The first factors to be entered into the model were the baseline measures, which were used as covariates. These were followed by inulin/placebo, order of conditions and then the interactions of all these variables. Daily diary data were analysed using Wilcoxon Signed Ranks test for non-parametric data. Significance levels were set at *p* < 0.05.

## 3. Results

### 3.1. Questions about the Previous Week and the Daily Diary

Data from these questionnaires was analysed using the Wilcoxon Signed Rank test for non-parametric data. There were no significant differences between the questions asking about the weeks prior to the inulin and placebo conditions. However, the daily diary scores showed some differences between inulin and placebo days.

#### 3.1.1. Feeling Happy

There was a significant effect of condition on how often participants felt happy (*z* = −2.403, *p* < 0.05): participants were more often happy when given the inulin than the placebo (see Table 1).

**Table 1:** Mean (and s.d.) scores for the two conditions (high scores = greater happiness, indigestion and hunger).

Variable	Inulin	Placebo
How often happy	2.64 (0.85)	2.34 (0.98)
Indigestion	0.17 (0.38)	0.40 (0.68)
Hunger	1.55 (0.53)	1.79 (0.91)

#### 3.1.2. Indigestion

There was a significant effect of condition on how often participants suffered with indigestion (*z* = −2.296, *p* < 0.05): participants suffered less indigestion when given the inulin than the placebo (see Table 1).

#### 3.1.3. Hunger

The effect of condition on how often participants felt hungry approached significance (*z* = −1.799, *p* = 0.072): participants felt less hungry when given the inulin than the placebo (see Table 1).

### 3.2. Mood Rating

The test-re-test reliability of the mood ratings (correlations between the baseline conditions on the two test days) ranged from 0.5 (anxiety after tasks) to 0.7 (alertness before tasks). There was no significant effect of inulin/placebo on mood scores (see Table 2).

**Table 2:** Mean mood scores (adjusted means; standard errors in parentheses) for the two conditions before and after the performance tasks (high scores = greater alertness, more positive affect and less anxiety).

Mood	Variable	Inulin	Placebo
Before cognitive tasks	Alertness	232.2 (9.5)	232.1 (9.5)
	Hedonic tone	182.2 (6.5)	181.3 (6.6)
	Anxiety	89.7 (2.9)	85.6 (3.4)
After cognitive tasks	Alertness	210.0 (8.4)	213.5 (8.8)
	Hedonic tone	174.4 (5.4)	174.9 (6.0)
	Anxiety	84.6 (2.7)	83.0 (3.0)

### 3.3. Memory Tasks

#### 3.3.1. Immediate Free Recall

The test-retest reliability for the number correct was 0.9, as was the test-retest reliability for the number of incorrect words. There was a significant effect of inulin/placebo with more words being recalled correctly (*F*(1,44) = 18.68, *p* < 0.001) and fewer incorrect words being written down (*F*(1,44) = 22.20, *p* < 0.001) in the inulin condition (see Table 3).

#### 3.3.2. Delayed Recall

The test-retest reliability for number of correct words was 0.6 and for number of incorrect words it was 0.5. There was a significant effect of inulin/placebo with more words being recalled correctly (*F*(1,44) = 6.72, *p* < 0.05) and fewer incorrect words being written down in the inulin condition (*F*(1,44) = 6.99, *p* < 0.05; see Table 3).

**Table 3:** Mean recall and recognition memory scores (adjusted means; standard errors in parentheses) for the inulin and placebo conditions.

Variable	Inulin	Placebo
Immediate recall: number correct	9.4 (0.4)	7.7 (0.4)
Immediate recall: number incorrect	0.8 (0.2)	1.9 (0.2)
Delayed recall: number correct	5.8 (0.4)	4.7 (0.4)
Delayed recall: number incorrect	1.2 (0.2)	2.0 (0.3)
Recognition memory: Percent correct responses	76.0 (1.6)	72.0 (1.7)
Recognition memory: mean RT (ms)	1053 (43)	951 (37)

#### 3.3.3. Delayed Recognition Memory

There was an effect of inulin/placebo condition with better accuracy (*F*(1,44) = 6.74, *p* < 0.05) but slower reaction times (*F*(1,44) = 3.99, *p* = 0.05) in the inulin condition (see Table 3).

#### 3.3.4. Logical Reasoning, Semantic Processing and Spatial Memory

The test-re-test reliability of the variables from these tasks ranged from 0.7 for the percentage of correct key presses in the spatial memory task to 0.9 for the number of trials completed in the semantic processing task. There no significant effects of inulin on these tasks (see Table 4).

**Table 4:** Mean memory scores for the two conditions (by memory task). Scores are the adjusted means from the Analysis of Covariance (standard errors in parentheses).

Task	Variable	Inulin	Placebo
Logical reasoning	Number of trials completed	49.7 (2.3)	54.6 (4.1)
	% Correct responses	79.9 (2.2)	79.2 (2.5)
Semantic processing	Number of trials completed	63.7 (2.0)	65.4 (1.9)
	% Correct responses	92.5 (1.8)	90.6 (1.0)
Spatial memory	Number of trials correct	1.58 (0.2)	1.63 (0.2)
	% Correct key presses	68.4 (3.0)	68.6 (2.9)

### 3.4. Psychomotor Tasks and Sustained Attention

The test-re-test reliability for variables from these tasks ranged from 0.5 for vigilance false alarms to 0.8 for simple reaction time. There were no significant effects on the simple and choice reaction time tasks. Similarly, there were no significant effects on the repeated digits vigilance task (see Table 5).

**Table 5:** Mean memory scores for the two conditions (by memory task). Score are the adjusted means from the Analyses of Covariance (standard errors in parentheses).

Task	Variable	Inulin	Placebo
Simple reaction time	Mean reaction time (rt) (ms)	358 (7.7)	365 (8.7)
Categoric search	Mean rt with no distracter (ms)	520 (7.6)	526 (7.3)
	Long responses (>800 ms)	11 (1.3)	12 (1.5)
Focused attention	Mean rt with no distracter (ms)	441 (8.7)	429 (9.1)
	Mean rt with distracters (ms)	438 (8.1)	435 (8.8)
	Long responses (>800 ms)	2.8 (0.8)	2.5 (0.8)
Vigilance	Mean rt (ms)	745 (14.5)	764 (16.3)
	Number of hits	16.2 (0.7)	16.0 (0.8)
	False alarms	11.1 (0.6)	10.9 (0.5)

## 4. Discussion

This preliminary study of the effects of oligofructose-enriched inulin on mood and performance demonstrated selective improvements followed ingestion of inulin. Episodic memory tasks, namely free recall and recognition memory, showed an improvement, whereas there were no significant effects of inulin on mood, psychomotor performance and selective and sustained attention. Larger doses of inulin may have different effects to the ones described here. This may reflect the increase in digestive symptoms which may be associated with impairments rather than benefits.

The effects on memory were apparent two hours after the ingestion of the inulin which rules out a prebiotic effect. One must ask what mechanism could underlie these acute effects of inulin. As well as being a prebiotic inulin has been shown to have a number of other physiological effects. For example, inulin improves lipid metabolism, increases both enzymatic and chemical protective functions in the gut and produces a range of enteroendocrine derived peptides that are known to create links between the gut and the brain [9]. Some of these, such as ghrelin, are known to improve learning and memory [25].

Inulin may also enhance glycaemic control by reducing post-prandial increases in serum glucose, delaying gastric emptying and slowing the entry of glucose into the blood stream [26]. Glucose has often been shown to have its strongest effects of episodic memory [27] and poor glucose tolerance is associated with impaired memory [28]. This is consistent with the findings obtained here and future research on oligofructose-enriched inulin should combine memory tests with measures of glucose metabolism. This should not only include levels of blood glucose but also measure glucose intolerance and insulin resistance, both of which have been shown to influence cognition [29,30]. Future research should also compare individual administration of oligofructose and inulin with the combined product which is favoured in commercial products.

## 5. Conclusions

There is a large literature on oligofructose-enriched inulin but little is known about the behavioural changes that follow ingestion of it. The aim of the present study was to provide initial data on the acute effects of consumption of oligofructose-enriched inulin. The results showed that after consumption of inulin volunteers felt happier, had less indigestion and were less hungry. The most consistent behavioural effects were an improvement of episodic memory (recall and recognition) after consumption of inulin. Further research is now needed to identify the mechanisms that underlie this effect.
